# Open Access gains attention in scholar communication

**DOI:** 10.1186/1476-4598-3-23

**Published:** 2004-09-06

**Authors:** Paul J Chiao, Christian Schmidt

**Affiliations:** 1 Molecular Cancer, BioMed Central Ltd, Middlesex House, 34-42 Cleveland Street, London W1T 4LB, UK

## 

Open Access is one of the attempts to maximize the exchange of information, and therefore benefits the scholar communication [1]. *Molecular Cancer *offers Open Access to all of its content, thereby providing a platform to present information to specialists and the public in order to further promote free exchange of ideas, concepts and findings in all fields of cancer-related biomedical science. All the published articles in the journal are determined by the peer review process.

Open Access has following broad benefits for science and the general public:

• All articles become freely and universally accessible online; so an author's work can be read by anyone at no cost.

• The authors hold copyright for their work and grant anyone the right to reproduce and disseminate the article, provided that it is correctly cited.

• A copy of the full text of each Open Access article is permanently archived in an online repository separate from the journal, such as PubMed Central, the US National Library of Medicine's full-text repository of life science literature, the repositories at the University of Potsdam in Germany, at INIST in France and in e-Depot, the National Library of the Netherlands' digital archive of all electronic publications.

• Authors are assured that their work is disseminated to the widest possible audience. This is accentuated by the authors being free to reproduce and distribute their work, for example by placing it on their institution's website. It has been suggested that free online articles are more highly cited because of their easier availability [2].

• The information available to researchers will not be limited by their library's budget, and the widespread availability of articles will enhance literature searching.

• The results of publicly funded research will be accessible to all interested readers and not just those with access to a library with a subscription. As such, Open Access could help to increase public interest in, and support of, research. Please note that this public accessibility may become a legal requirement in the USA if the proposed Public Access to Science Act is made law [3].

• A country's economy will not influence its scientists' ability to access articles because resource-poor countries (and institutions) will be able to read the same material as wealthier ones, although creating access to the internet is another matter.

*Molecular Cancer *published a number of interesting papers, and the list of the top ten most accessed articles is available at . All papers accepted by *Molecular Cancer *appear as 'accepted manuscript' on the web pages and are subsequently included in PubMed. A fully formatted portable document file is available approximately two to three weeks after acceptance along with a web-version of the article.

The on-line publication, to the exclusion of print, has many advantages: Coloured pictures can be presented along with large sets of supporting data (movies, tables, pictures, et cetera) without additional charges. In addition, the on-line submission process allows a fast and effective handling of papers and allows authors to check the status of their submitted manuscript(s). There is no limitation in space, but concise papers are more likely to be read.

The peer review policy, described in [4], ensures a fair evaluation of the work. We wish to thank our authors for sending their work to *Molecular Cancer*, all members of the editorial board and the reviewers for their ongoing support for Open Access publishing and for aiming higher standards for *Molecular Cancer*.

The acceptance rate of *Molecular Cancer *did not change significantly, compared to the last report [4]. One out of three incoming articles are accepted for publication at *Molecular Cance*r after revisions. In addition to indexing in PubMed, PubMed Central and other search engines, *Molecular Cancer *is working closely with the Institute for Scientific Information to ensure that citation analysis of our articles will be available.

## Competing interests

PJC is Editor-in-Chief and CS is Deputy Editor of this journal. Both do not receive any remuneration for their efforts but they are exempted from the article processing fee for this journal.
